# Effects of CDX2 on prognosis and chemotherapy responsiveness in mismatch repair‐deficient colorectal cancer

**DOI:** 10.1002/bjs5.91

**Published:** 2018-07-24

**Authors:** É. J. Ryan, B. Creavin, Y. L. Khaw, M. E. Kelly, H. M. Mohan, R. Geraghty, E. J. Ryan, R. Kennelly, A. Hanly, S. T. Martin, D. Fennelly, R. McDermott, D. Gibbons, P. R O'Connell, K. Sheahan, D. C. Winter

**Affiliations:** ^1^ Department of Surgery, St Vincent's University Hospital Dublin Ireland; ^2^ Centre for Colorectal Disease, St Vincent's University Hospital Dublin Ireland; ^3^ Department of Histopathology, St Vincent's University Hospital Dublin Ireland; ^4^ Department of Oncology, St Vincent's University Hospital Dublin Ireland; ^5^ School of Medicine and Medical Sciences University College Dublin Dublin Ireland

## Abstract

**Background:**

Caudal‐related homeobox transcription factor 2 (CDX2) is an intestine‐specific transcription factor implicated in tumour differentiation, proliferation, cell adhesion and migration. Negative CDX2 status (CDX2−) is associated with worse prognosis in colorectal cancer and may identify high‐risk stage II disease that benefits from adjuvant chemotherapy. This observational study investigated whether CDX2− is associated with prognosis or response to chemotherapy in the mismatch repair‐deficient (dMMR) phenotype of colorectal cancer.

**Methods:**

Patients with resectable dMMR colorectal cancer were eligible for inclusion. The prognostic and predictive value of CDX2 expression on the presence of lymph node metastasis (LNM) and survival was investigated. CDX2 status was determined via immunohistochemistry using the Leica Bond™ CDX2 (clone EP25) ready‐to‐use primary antibody.

**Results:**

Some 235 of 238 consecutive dMMR tumours were assessed for CDX2 status. CDX2− was observed in 15·7 per cent of colorectal cancer. Interobserver agreement was excellent (*κ* = 0·863; *P* < 0·001). CDX2− was significantly associated with female sex, increased size, advanced stage, worse conventional and poorly differentiated cluster (PDC) grade, mucinous morphology, perineural and lymphovascular invasion, and pN status (all *P* ≤ 0·038). CDX2− was not associated with LNM or survival in multivariable analysis. Independent predictors of LNM were PDC grade (odds ratio (OR) 4·12, 95 per cent c.i. 1·76 to 9·63; *P* = 0·001) and extramural venous invasion (OR 3·79, 1·62 to 8·85; *P* = 0·002). Budding (hazard ratio (HR) 2·79, 95 per cent c.i. 1·60 to 4·87; *P* < 0·001), pT status (HR 3·59, 1·29 to 10·01; *P* = 0·015) and adjuvant chemotherapy (HR 2·07, 1·15 to 3·74; *P* = 0·016) were independently associated with worse disease‐free survival.

**Conclusion:**

CDX2− does not confer a worse prognosis in the dMMR phenotype of colorectal cancer. The MMR status of patients with colorectal cancer should be determined before assessing CDX2 status.

## Introduction

Although molecular profiles of colorectal cancers have been characterized in detail in recent years^1^, for practical purposes there are two main molecular subtypes: those with microsatellite instability (MSI) (15–20 per cent) where errors in replication go unchecked due to deficient DNA mismatch repair (dMMR), and mismatch repair (MMR)‐proficient (pMMR) tumours (80–85 per cent)^2^. The majority of dMMR cancer is sporadic and caused by *MLH1* promoter hypermethylation, occurring on a background of global gene promoter hypermethylation known as the CpG island methylator phenotype (CIMP)^3^. Such tumours are usually diagnosed at advanced age with a female preponderance, are associated with *BRAF* mutation, and originate from sessile serrated lesions (SSLs)^2^. Between 3 and 5 per cent of all colorectal cancers, however, are thought to be due to constitutional mutations in the MMR genes (Lynch syndrome)^4^. Aside from helping to identify patients with this syndrome^5^, interest in determining MMR status has grown as a result of a role in prognostic stratification^6^ ^7^, and as a predictor of response to chemotherapy^8^ and novel immunotherapies^9^ ^10^.

CDX2 is a homeobox gene/intestine‐specific transcription factor essential for intestinal development and differentiation^11^. It is restricted to the adult small intestine and colon, and is an important biomarker of mature colonic epithelial tissue. It has been proposed to govern diverse processes such as cell proliferation, adhesion and migration, and tumorigenesis, and may have both oncogenic and tumour‐suppressing potential^12^. Colorectal cancer with negative CDX2 status (CDX2−) is associated with an increased likelihood of aggressive features such as lymph node metastasis (LNM), poor differentiation, lymphovascular (LVI), perineural (PNI) and extramural vascular (EMVI) invasion, *BRAF* mutation and CIMP^12^. CDX2− has been shown to be associated with a worse disease‐free survival (DFS) in colorectal cancer^13^. It has been hypothesized that the prognostic effect of CDX2− could be explained by its capacity to function as a single biomarker for many biological risk factors, under the common motif of an immature stem cell phenotype. Moreover, CDX2 status may identify high‐risk stage II disease (CDX2− disease) that has a high risk of recurrence and in which the hazards of chemotherapy may be offset by a survival benefit^13^.

Several studies have shown that dMMR colorectal cancer has a better prognosis than pMMR colorectal cancer^6^ ^7^, whereas experimental and clinical evidence suggests a reduced response to 5‐fluorouracil (5‐FU)‐based adjuvant chemotherapy in dMMR tumours^8^ ^14^. This has led to the recommendation that MMR testing should be considered for all patients with stage II disease^15^. Loss or downregulation of CDX2 expression, however, occurs more frequently in the dMMR phenotype (approximately 15 per cent) than in colorectal tumours overall (less than 5 per cent)^12^ ^13^.

The aim of the present study was to investigate the apparently counterintuitive association of dMMR colorectal cancer, with its reported good prognosis and resistance to 5‐FU, with CDX2− disease, a phenotype conferring an adverse prognosis and a benefit from 5‐FU.

## Methods

This was a single‐centre observational study of patients tested for dMMR between 1 January 2005 and 1 January 2015. Patients with primary resectable colorectal cancer were retrieved from an institutional database. All had a confirmed histological diagnosis of colorectal cancer and had undergone surgical resection. Patient selection for colorectal cancer resection was in accordance with the Royal College of Surgeons in Ireland guidelines, and all patients were discussed at a multidisciplinary meeting^16^. The study protocol was reviewed and approved by St Vincent's University Hospital research and ethics committee. A full description of all methods is available in *Appendix* S1 (supporting information).

### Mismatch repair status

MMR status was assessed using immunohistochemistry (IHC) for MMR proteins, human mutL homologue (hMLH) 1 (clone G168‐728; BD Biosciences®, Franklin Lakes, New Jersey, USA), human postmeiotic segregation (hPMS) 2 (clone A16‐4; BD Biosciences®), human mutS homologue (hMSH) 2 (clone FE11; Calbiochem®, San Diego, California, USA) and hMSH6 (clone 44; BD Biosciences®), as described previously^17^.

### CDX2 status

Formalin‐fixed, paraffin‐embedded tissue sections were stained with Leica Bond™ CDX2 (clone EP25; Leica Biosystems, Wetzler, Germany) ready‐to‐use primary antibody. IHC was performed on the automated Leica BOND™ platform. Antigen retrieval with Leica Bond™ ER2 solution (30 min) was performed with an antibody incubation time of 15 min. Visualization of the antibody–antigen reaction was via the Leica Bond™ Polymer Refine Detection system (Leica Biosystems). Percentage CDX2 expression and the intensity of the immunoreaction were estimated in each case. To assess CDX2 status, the scoring system developed by Dalerba and colleagues^13^ was used. Two consultant histopathologists reviewed all available specimens. When there was disagreement, a third histopathologist decided on the CDX2 status.

### Follow‐up and outcomes

The prognostic and predictive value of CDX2 expression on the presence of LNM and survival was investigated. Clinical follow‐up was at 6 weeks and 3–6‐monthly intervals thereafter, and included endoscopic assessment. For 3 years, all patients had 6‐monthly CT of the thorax, abdomen and pelvis. Survival and recurrence were recorded in a prospectively developed institutional colorectal cancer database. Locoregional recurrence was defined as recurrence, either biopsy‐proven or with convincing imaging and concurrent increase in tumour markers, located in the abdominal or pelvic nodes, at the anastomotic site or rectal stump, or in the peritoneum, presacral area or retroperitoneum as a soft tissue mass. Mortality status and cause of death were confirmed from data obtained from the National Cancer Registry in Cork and the General Registrar's Office in Dublin, Ireland. Primary care physicians were contacted as necessary to complete survival data if cause of death was unclear. The last date of follow‐up was 31 March 2016.

### Statistical analysis

All results were analysed using IBM SPSS® Statistics version 21.0 (2012) for Mac OS® (IBM, Armonk, New York, USA) and GraphPad Prism® version 7.0 (2016) for Mac OS® (GraphPad Software, San Diego, California, USA). Cohen's κ coefficient was used to test interobserver reliability. The statistical association between CDX2 status and the various histological parameters was investigated using Fisher's exact test for categorical data. Student's *t* test and the Mann–Whitney *U* test were used for continuous variables as appropriate. Variables with *P* < 0·200 in univariable analysis were included in multivariable analysis. The Hosmer–Lemeshow test was used to assess the goodness‐of‐fit of the binary logistic regression model. Kaplan–Meier curves, log rank test and Cox regression were used to associate survival with CDX2 status, and the various molecular and pathological characteristics were expressed as hazard ratios (HRs) with 95 per cent confidence intervals. All tests of significance were two‐tailed, with *P* < 0·050 indicating statistical significance.

## Results

Some 238 patients with primary dMMR cancers were identified. No further tissue was available for CDX2 analysis on three dMMR slides that had been referred from external institutions for MMR testing. Therefore, only 235 patients and tumours were available for CDX2 assessment.

The median length of follow‐up for all 238 patients with dMMR cancers was 45 (range 3–144) months. The mean(s.d.) age of the cohort was 71·2(13·8) (range 23–97) years. The majority of patients were women (65·5 per cent), and tumours occurred most frequently (84·0 per cent) in the right colon (*Table* S1, supporting information). Of 204 patients with MLH1‐ and MLH1/PMS2‐deficient tumours, 199 (97·5 per cent) underwent reflex *BRAF* mutational testing to screen for Lynch syndrome, of whom 58 (29·1 per cent) were *BRAF* wild‐type. Twenty‐five patients with dMMR had a confirmed Lynch syndrome constitutional mutation. CDX2− was observed in 15·7 per cent of dMMR colorectal cancers (*Fig.* S1, supporting information). Agreement between observers was excellent (κ = 0·863, *P* < 0·001) with regard to the final assessment of CDX2 status.

### Comparisons with other demographic and histopathological features

*Table* 1 compares basic patient and tumour characteristics between patients with CDX2− and those with CDX2+ tumours. CDX2− was significantly associated with patient sex (*P* = 0·037), tumour size (*P* = 0·002) and AJCC (seventh edition) stage (*P* = 0·019). With regard to histopathological features, CDX2 status was associated with WHO grade (*P* < 0·001), poorly differentiated cluster (PDC) grade (*P* = 0·012), mucinous type (*P* = 0·001), PNI (*P* = 0·038), LVI (*P* = 0·002) and pN category (*P* = 0·016) (*Table* 2).

**Table 1:** Comparison of patient demographics and general pathological features according to CDX2 status in colorectal cancer with mismatch repair‐deficient phenotype

	*n*	CDX2− (*n* = 37)	CDX2+ (*n* = 198)	*P* †
Age (years)*	235	71·5(15·1) (23–90)	71·4(13·4) (28–97)	0·603‡
Sex				0·037
F	153	30 (19·6)	123 (80·4)	
M	82	7 (8·5)	75 (91·5)	
Tumour size (cm)*		70·5(25·4) (30–110)	57·6(28·9) (5–160)	0·002§
Site				0·227
Right	197	34 (17·3)	163 (82·7)	
Left	38	3 (7·9)	35 (92·1)	
AJCC TNM stage				0·019
1–2	166	20 (12·0)	146 (88)	
3–4	69	17 (24·6)	52 (75·4)	

Values in parentheses are percentages unless indicated otherwise;

* values are mean(s.d.) (range). CDX, caudal‐related homeobox transcription factor.

† Fisher's exact test, except

‡ Student's *t* test and

§ Mann–Whitney *U* test.

**Table 2:** Comparison of detailed histopathological and molecular features according to CDX2 status colorectal cancer with mismatch repair‐deficient phenotype

	*n*	CDX2− (*n* = 37)	CDX2+ (*n* = 198)	*P* *
*BRAF*				0·837
Wild‐type	62	9 (15)	53 (85)	
Positive	143	23 (16·1)	120 (83·9)	
WHO grade				< 0·001
1–2	149	11 (7·4)	138 (92·6)	
3–4	86	26 (30)	60 (70)	
Tumour budding				0·327
No	130	10 (7·7)	120 (92·3)	
Yes	37	5 (14)	32 (86)	
PDC grade				0·012
Low	126	7 (5·6)	119 (94·4)	
High	41	8 (20)	33 (80)	
Mucinous (%)				0·001
≤ 50	197	37 (18·8)	160 (81·2)	
> 50	38	0 (0)	38 (100)	
Signet ring				0·512
No	216	33 (15·3)	183 (84·7)	
Yes	19	4 (21)	15 (79)	
LVI				0·002
No	106	8 (7·5)	98 (92·5)	
Yes	129	29 (22·5)	100 (77·5)	
PNI				0·038
No	201	27 (13·4)	174 (86·6)	
Yes	34	10 (29)	24 (71)	
EMVI				0·252
No	159	22 (13·8)	137 (86·2)	
Yes	75	15 (20·0)	60 (80)	
Type of margin				0·285
Expansile	115	15 (13·0)	100 (87·0)	
Infiltrative	119	22 (18·5)	97 (81·5)	
pT category				0·054
pT1–2	40	2 (5)	38 (95)	
pT3–4	195	35 (17·9)	160 (82·1)	
pN category				0·016
pN0	166	20 (12·0)	146 (88·0)	
pN1–2	69	17 (25)	52 (75)	

Values in parentheses are percentages. CDX, caudal‐related homeobox transcription factor; PDC, poorly differentiated cluster; LVI, lymphovascular invasion; PNI, perineural invasion; EMVI, extramural vascular invasion.

* Fisher's exact test.

### Risk factors for lymph node metastasis

LNM was observed in 29·4 per cent of tumours (70 of 238) in the entire dMMR cohort. Of the 235 tumours that were assessed for CDX2 status, the LNM rate was 46 per cent (17 of 37) in CDX2− tumours and 26·3 per cent (52 of 198) in CDX2+ tumours. CDX2 status (*P* = 0·018), WHO grade (*P* = 0·002), tumour budding (*P* < 0·001), PDC grade (*P* < 0·001), LVI (*P* < 0·001), PNI (*P* < 0·001), EMVI (*P* < 0·001), type of margin (*P* = 0·001) and pT category (*P* = 0·006) were associated with LNM in univariable analysis (*Table* 3). The only independent predictors of LNM, however, were PDC grade (OR 4·12, 95 per cent c.i. 1·76 to 9·63; *P* = 0·011) and EMVI (OR 3·79, 1·62 to 8·85; *P* = 0·002) (*Table* 3).

**Table 3:** Binary logistic regression analysis of histopathological predictors of lymph node metastasis

	Univariableanalysis		Multivariableanalysis	
	Odds ratio	*P*	Odds ratio	*P*
Age (years)	1·00 (0·98, 1·02)	0·712		
Male sex (*versus* female sex)	0·56 (0·30, 1·05)	0·069		
Left side (*versus* right side)	0·97 (0·45, 2·09)	0·945		
CDX2− (*versus* CDX2+)	2·39 (1·16, 4·90)	0·018		
*BRAF*+ (*versus* wild‐type)	1·08 (0·57, 2·05)	0·822		
High WHO grade (*versus* low grade)	2·50 (1·41, 4·43)	0·002		
High budding (*versus* low budding)	5·18 (2·35, 11·43)	< 0·001		
High PDC grade (*versus* low grade)	6·52 (2·98, 14·28)	< 0·001	4·12 (1·76, 9·63)	0·001
LVI present (*versus* absent)	4·44 (2·33, 8·47)	< 0·001		
PNI present (*versus* absent)	5·11 (2·38, 10·96)	< 0·001		
EMVI present (*versus* absent)	6·09 (3·31, 11·23)	< 0·001	3·79 (1·62, 8·85)	0·002
Infiltrative margin present (*versus* absent)	2·81 (1·56, 5·07)	0·001		
High pT category (*versus* low category)	4·50 (1·54, 13·18)	0·006		

Values in parentheses are 95 per cent confidence intervals. CDX, caudal‐related homeobox transcription factor; PDC, poorly differentiated cluster; LVI, lymphovascular invasion; PNI, perineural invasion; EMVI, extramural vascular invasion.

### Survival

**Figure 1:**
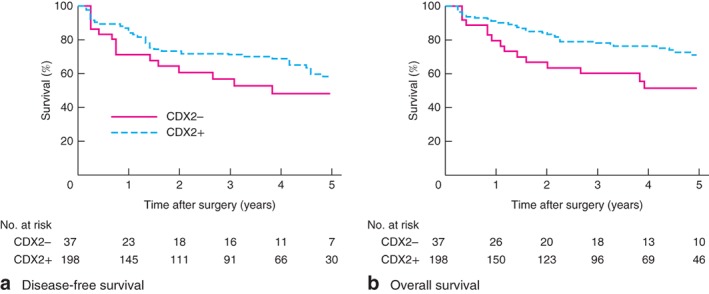
Kaplan–Meier curves of **a** disease‐free and **b** overall survival in patients with the mismatch repair‐deficient (dMMR) phenotype of colorectal cancer according to CDX2 status. **a** *P* = 0·198, **b** *P* = 0·009 (log rank test)

Some 40 patients (16·8 per cent) had recurrent cancer, most frequently locoregional recurrence (37 patients, 93 per cent); all three distant recurrences were to the lung. There was no significant difference in 5‐year disease‐free survival according to CDX2 status (*Fig*. 1 *a*). CDX2− carcinomas were associated with reduced 3‐year (60 per cent *versus* 77·8 per cent for CDX+ tumours; *P* = 0·018) and 5‐year (51·0 *versus* 70·1 per cent respectively; *P* = 0·009) overall survival (*Fig*. 1 *b*). However, the prognostic effect of CDX2 expression for overall survival in dMMR colorectal cancer was not independent of stage (*Fig.* S2, supporting information).

Chemotherapy was not associated with an improvement in 3‐year disease‐free survival once adjusted for stage (*Fig.* S3, supporting information). There was no difference in response based on CDX2 expression. Using Cox regression, treatment with adjuvant chemotherapy was associated with reduced disease‐free survival (HR 2·06, 95 per cent c.i. 1·25 to 3·38; *P* = 0·004) in univariable analysis. This effect remained significant in the multivariable model (HR 2·07, 1·15 to 3·74; *P* = 0·016) (*Table* 4). Other variables associated with worse disease‐free survival in multivariable analysis were pT category (HR 3·59, 1·29 to 10·01; *P* = 0·015) and budding (HR 2·79, 1·60 to 4·87; *P* < 0·001). Only PNI (HR 2·74, 1·27 to 7·20; *P* = 0·010) and the presence of LNM (HR 2·13, 1·10 to 4·15; *P* = 0·026) were independently associated with worse overall survival (*Table* 5).

**Table 4:** Cox regression analysis of disease‐free survival in patients with colorectal cancer with mismatch repair‐deficient phenotype

	Univariable analysis		Multivariable analysis	
	Hazard ratio	*P*	Hazard ratio	*P*
Age (years)	0·99(0·97, 1·00)	0·074		
Male sex (*versus* female sex)	1·11 (0·72, 1·73)	0·636		
Left side (*versus* right side)	1·40 (0·83, 2·36)	0·215		
CDX2− (*versus* CDX2+)	1·42 (0·83, 2·41)	0·198		
*BRAF*+ (*versus* wild‐type)	1·06 (0·63, 1·72)	0·829		
High WHO grade (*versus* low grade)	1·57 (1·03, 2·41)	0·036		
High budding (*versus* low budding)	2·91 (1·68, 5·04)	< 0·001	2·79 (1·60, 4·87)	< 0·001
High PDC grade (*versus* low grade)	2·42 (1·39, 4·20)	0·003		
LVI present (*versus* absent)	1·70 (1·09, 2·66)	0·020		
PNI present (*versus* absent)	2·44 (1·46, 4·10)	0·001		
EMVI present (*versus* absent)	2·78 (1·82, 4·26)	< 0·001		
Infiltrative margin present (*versus* absent)	2·39 (1·52, 3·74)	< 0·001		
High pT category (*versus* low category)	4·18 (1·69, 10·31)	0·002	3·59 (1·29, 10·01)	0·015
Positive nodal status (*versus* negative status)	2·45 (1·60, 3·77)	< 0·001		
Adjuvant chemotherapy (*versus* surgery alone)	2·06 (1·25, 3·38)	0·004	2·07 (1·15, 3·74)	0·016

Values in parentheses are 95 per cent confidence intervals. CDX, caudal‐related homeobox transcription factor; PDC, poorly differentiated cluster; LVI, lymphovascular invasion; PNI, perineural invasion; EMVI, extramural vascular invasion. Schoenfeld residual test: *P* = 0·427.

**Table 5:** Cox regression analysis of overall survival in patients with colorectal cancer with mismatch repair‐deficient phenotype

	Univariable analysis		Multivariable analysis	
	Hazard ratio	*P*	Hazard ratio	*P*
Age (years)	0·99 (0·97, 1·01)	0·989		
Male sex (*versus* female sex)	1·07 (0·65, 1·76)	0·806		
Left side (*versus* right side)	1·24 (0·68, 2·27)	0·490		
CDX2− (*versus* CDX2+)	2·03 (1·18, 3·49)	0·010		
*BRAF*+ (*versus* wild‐type)	1·40 (0·74, 2·66)	0·303		
High WHO grade (*versus* low grade)	1·57 (1·03, 2·41)	0·036		
High budding (*versus* low budding)	2·91 (1·68, 5·04)	< 0·001		
High PDC grade (*versus* low grade)	2·42 (1·39, 4·20)	0·003		
LVI present (*versus* absent)	1·70 (1·09, 2·66)	0·020		
PNI present (*versus* absent)	2·44 (1·46, 4·10)	0·001	2·74 (1·27, 7·20)	0·010
EMVI present (*versus* absent)	2·78 (1·82, 4·26)	< 0·001		
Infiltrative margin present (*versus* absent)	2·39 (1·52, 3·74)	< 0·001		
High pT category (*versus* low category)	4·18 (1·69, 10·31)	0·002		
Positive nodal status (*versus* negative status)	2·45 (1·60, 3·77)	< 0·001	2·13 (1·10, 4·15)	0·026
Adjuvant chemotherapy (*versus* surgery alone)	0·80 (0·42, 1·51)	0·487		

Values in parentheses are 95 per cent confidence intervals. CDX, caudal‐related homeobox transcription factor; PDC, poorly differentiated cluster; LVI, lymphovascular invasion; PNI, perineural invasion; EMVI, extramural vascular invasion.

## Discussion

In this study CDX2− status in dMMR colorectal cancer was associated with a number of adverse prognostic variables such as stage, conventional WHO grade, PDC grade, PNI and LVI, but not with survival. This shows that dMMR colorectal cancer is a heterogonous phenotype with different prognostic factors compared with pMMR disease. CDX2 testing does not appear to provide additional prognostic or predictive information in the management of dMMR tumours. In the authors' opinion, colorectal cancer should be tested for MMR status first, and CDX2 testing should be considered only when the tumour is pMMR.

The recent international consensus molecular subtype (CMS) classification divides colorectal cancer into four CMS groups based on gene expression profiling^1^, and emerging evidence^18^ suggests that these have distinct immune orientation. Initial studies^19^, ^20^, ^21^ have found an association between lack of CDX2 expression and reduced survival in dMMR colorectal cancer (CMS1/immune subtype). Some studies^22^, ^23^, ^24^ have reported discordant results. Olsen and colleagues^23^ found that low CDX2 protein or mRNA expression was not associated with recurrence risk in a study of 119 patients with colorectal cancer, including 44 dMMR tumours. In a study of 469 colorectal cancers including 81 dMMR tumours, Pilati and co‐workers^22^ demonstrated that although CDX2− identified a group of patients with a particularly poor prognosis both globally and in the CMS4 (stem cell‐related/mesenchymal) group, it was not associated with a worse prognosis within the CMS1 subtype. More recently, Neumann *et al*.^24^ found, in a cohort of 503 patients with colorectal cancer (FIRE‐3) and in a case–control collection of 50 right‐sided tumours with and without synchronous distant metastasis, that CDX2 was not an independent prognostic biomarker in colorectal cancer. They found that the prognostic impact of CDX2 depended on the MMR and *BRAF* mutational status of these tumours. The present study has validated these findings in a large cohort of dMMR colorectal cancer.

Recently, Fessler and colleagues^25^ showed that transforming growth factor (TGF) β stimulation in an SSL model strongly reduced CDX2 expression, downregulated the expression of genes associated with colorectal intraepithelial neoplasia and induced the expression of mesenchymal marker genes. Thus, SSLs may progress to either CMS1 (dMMR/MSI) or CMS4, depending on the level of TGFβ signalling activity. The assumed better prognosis of dMMR colorectal cancer may reflect the marked immune cell infiltration of its tumour microenvironment^26^. TGFβ in the tumour microenvironment also stimulates the recruitment of immune cells, which have a significant role in the regulation of tumour progression, while directly inhibiting their antitumour effector functions^27^. Therefore, in poorly differentiated tumours there may be two distinct scenarios. In CDX2− CMS4 tumours TGFβ may polarize infiltrating lymphocytes towards a more regulatory phenotype, with less potent antitumour effector functions^27^, whereas in CDX2− CMS1 tumours the absence of TGFβ may result in a more robust cytotoxic T‐cell response^18^. In the present study, tumour budding, associated with both TGFβ and epithelial mesenchymal transition^28^, was a stage‐independent predictor of worse outcome.

In some studies the supposed survival advantage of dMMR seems to be independent of tumour stage^29^ ^30^, whereas in others it appears to be confined to stage II^31^ or stage III^32^ disease, although these data are likely confounded by the effect of adjuvant therapy^33^. Preclinical evidence suggests that dMMR might be one mechanism for tumour resistance to 5‐FU^14^ and, although results were conflicting, numerous clinical data suggest a possible reduced response to 5‐FU in dMMR colorectal cancer^8^. In contrast, dMMR tumour cells appear sensitive to oxaliplatin^34^, and retrospective analyses of a number of large clinical trials suggest that treatment with FOLFOX (folinic acid–5‐FU–oxaliplatin) is superior to 5‐FU in dMMR colorectal cancer^35^, ^36^, ^37^. In the present study, neither therapy demonstrated a survival advantage above that of surgery alone. Unlike the findings of Dalerba and colleauges^13^, there was no difference in response to chemotherapy based on CDX2 expression.

Limitations of this study include the absence of a comparator group of patients with stage‐ and aged‐matched pMMR colorectal cancer and the retrospective design, which may have led to selection bias. Evolving knowledge regarding the heterogeneity of colorectal cancer poses questions regarding the appropriateness of controls. Findings arising from colorectal cancer trials of novel therapies, and prognostic and predictive biomarkers, may not be applicable to all tumours.

## Supporting information


**Appendix S1.** Detailed methods
