# Supplementary material for: Effects of CDX2 on prognosis and chemotherapy responsiveness in mismatch repair‐deficient colorectal cancer
Source: BJS Open. 2018 Jul 24;2(6):456–63. doi: 10.1002/bjs5.91 (PMC6253792; doi:10.1002/bjs5.91)
Supplement: Supplementary file 1 — Appendix S1. Detailed methods [file BJS5-2-456-s001.docx]

**BJS5_91**

**Effects of CDX2 on prognosis and chemotherapy responsiveness in mismatch repair-deficient colorectal cancer**

**É. J. Ryan, B. Creavin, Y. L. Khaw, M. E. Kelly, H. M. Mohan, R. Geraghty, E. J. Ryan, R. Kennelly, A. Hanly, S. T. Martin, D. Fennelly, R. McDermott, D. Gibbons, P. R O’Connell, K. Sheahan and D. C. Winter**

**Appendix S1** Detailed methods

**Study Design**

This current study is a single-centre observational study of a series of 2086 patients prospectively tested for dMMR over a 10-year period (2005 to 2015). Patients with primary CRC were identified from a prospectively maintained database. All patients included on this database had a confirmed diagnosis of CRC and underwent surgical resection. Patient selection for CRC resection was in accordance with the Royal College of Surgeons in Ireland guidelines and all patients were discussed at the institutional multidisciplinary meeting. ^1^ Only patients with primary CRC that had subsequent resection were included.

**Method Of Staging**

Staging was performed in accordance with the American Joint Committee on Cancer (AJCC), version 7 Guidelines. ^2^ Clinical staging was determined by CT thorax, abdomen and pelvis for colorectal cancers with additional pelvic MRI scan for rectal cancer. Pathological analysis using a standardized reporting template was performed on resected specimens and staged according to AJCC criteria. ^2^

**Method Of Assessing MMR Status**

MMR status was assessed using immunohistochemistry (IHC) for mismatch repair proteins, hMLH1 (BD Bioscience, clone G168-728), hPMS2 (BD Biosciences, clone A16-4, hMSH2 (Calbiochem, clone FE11) and hMSH6 (BD Biosciences, clone 44) as described by Mohan et al.^3^ Four micron sections were cut from archival formalin fixed paraffin embedded (FFPE) blocks and incubated in a 60*C oven for 2 hours. Automated immunohistochemistry was performed on the BOND instrument (Leica). In the protocol antigen retrieval was performed using Bond Epitope Retrieval Solution 2 for 30 minutes. Leica ready to use CDX2 antibody (clone EP25) was incubated on the sections for 15 minutes at room temperature, Visualisation of the antibody antigen reaction was via the Leica Bond Polymer Refine Detection system.

Nuclear staining in any area of the tumor was classified as showing no loss of the mismatch repair proteins. Tumors showing complete loss of nuclear staining of the mismatch repair proteins in the entire tumor with concurrent positive staining of nuclei of non-neoplastic cells was classified as having loss of expression of that mismatch repair protein.

**Method of Assessing CDX2 Expression**

FFPE tissue sections were stained with Leica Bond CDX2 (clone EP25) ready to use primary antibody. The presence of the CDX2 antigen in the tissue sections was visualized using the BOND automated immunohistochemistry staining instrument (Leica). Parrafin wax was removed from the sections at a high temperature using Bond Dewax solution (Leica). Antigen retrieval was then performed using Bond Epitope Retrieval Solution 2 (Leica) for 20 minutes. This solution contains an EDTA buffer and surfactant, PH 8.9-9.1. Visualisation of the antibody antigen reaction was via the Leica Bond Polymer Refine Detection system. This utilises a novel controlled polymerisation technology to prepare polymeric HRP-linker antibody conjugates. The detection system avoids the use of streptavidin and biotin, and therefore eliminates non-specific staining as a result of endogenous biotin. The Leica CDX2 (E25) primary antibody has been specifically optimised for use with the Bond Polymer Refine Detection. The steps involved in the detection system are as follows: The specimen is incubated with hydrogen peroxide to quench endogenous activity. Leica ready to use CDX2 antibody (clone EP25) is applied for 15 minutes at room temperature. The post primary IgG linker reagent localises to the bound antigen antibody complex. The Poly-HRP IgG reagent localises to the linker reagent. The substrate chromagen, 3-3-Diaminobenzidine tetrahydrochloriide hydrate (DAB), visualises the complex via a brown precipitate. Haematoxylin (blue) counterstaining allows the visualisation of cell nuclei.

We used the scoring system developed by Dalerba and colleagues, with some modifications.^4^ Two independent investigators used the same criteria (S2) to independently score CDX2 protein expression levels in 235 FFPE sections from primary CRC. The concordance between the two observers was analysed using contingency tables to calculate the Cohen’s Kappa Index. . The results showed a near-perfect agreement (k = 0.863, p < 0.000) with regard to the final assessment of the patients’ CDX2 status according to the observations of the two consultant observers (S3)

We stratified tumours based on percentage expression of CDX2 and intensity of immunoreaction. (S2) We stratified CRCs in two categories: CDX2 negative tumours (CDX2neg) or CDX2 positive (CDX2pos) tumours. A percentage CDX2 expression was estimated in each case during an exploratory analysis and the strength of staining was measured as either absent (if no CDX2 expression evident), weak, moderate or strong.

We scored as CDX2neg (S2, Panels A & B) all tumors whose malignant epithelial component either completely lacked CDX2 expression or showed faint nuclear expression in a minority (≤ 20%) of malignant epithelial cells, a feature observed in 15.7% (n = 37/235) of dMMR CRCs. Tumors scored as CDX2neg fell into two staining patterns: a) complete lack of CDX2 expression (Score 0), observed in 5.9% (n = 14/235) of cases (S2, Panel A); b) scattered and faint nuclear expression in a minority fraction of cancer cells (Score 0.5), observed in 9.8% (n = 23/235) of cases (S2, Panel B).

We scored as CDX2pos (S2, Panels C & D) all tumors whose malignant epithelial component displayed widespread (>20%) nuclear expression of CDX2, a feature observed in 84.3% (n = 198/235) of dMMR CRC. Tumors scored as CDX2pos also fell into two staining patterns: a) moderate/strong staining in >20% but <100% of cancer cells (Score 2), observed in 56.9% (n = 58/235) of cases (S2, Panel C); b) strong staining in all cancer cells (Score 3), observed in 24.7% (n = 140/235) of dMMR cases (S2, Panel D).

**Method Of Assessing Anatomic Site**

Anatomic site was documented by measurement from known landmarks according to general guidelines defining colonic topography. In multivariable analysis anatomic site was classified as being right Sided (includes caecum, ascending colon, transverse colon and splenic flexure) or left sided (descending colon, sigmoid and rectum).

**Method Of Assessing Tumor Size**

Measurement on gross pathologic examination was considered the definitive determination of tumor size.

**Method Of Assessing Conventional WHO Grade**

For each case, histological grading based on glandular differentiation and PDC grade was performed on H&E stained sections. The median number of slides examined was 6 (range 2-11). Histological grading based on glandular differentiation was assessed according to WHO criteria. ^5^ Percentages of glandular and non-glandular components in each tumour were estimated. Each case was graded as follows: i) well differentiated – at least 95% glandular differentiation, ii) moderately differentiated – 5-95% glandular differentiation, iii) poorly differentiated – more than 50% solid / undifferentiated areas, iv) undifferentiated – less than 5% glandular differentiation. In multivariable analysis we grouped grades 1 and 2 as “low-grade” and grades 3 and 4 as “high-grade” CRCs as proposed in the fourth edition of the WHO classification. ^6^

**Method Of Assessing WHO Special Types**

Each component was classified into the following WHO growth ^6^ patterns and percentage of total tumour volume estimated: i) conventional – recognisable gland formation, ii) mucinous: Extracellular mucin production and associated malignant epithelium, iii) mucin-rich signet ring (tumour cells with prominent intracytoplasmic mucin) with abundant extracellular mucin, iv) mucin-poor signet ring (signet rings with minimal extracellular mucin and without glandular differentiation), v) solid - no recognisable glandular differentiation and includes a similar growth pattern within mucinous areas.

When a growth pattern occupied more than 50% of the total tumour volume, the tumour was subclassified based on the WHO definitions of tumours of special types ^6^, with some modifications: i) Conventional adenocarcinoma – tumours without a dominant pattern of more than 50% total volume, ii) mucinous adenocarcinoma – tumors with more than 50% tumour with extracellular mucin, excluding mucin-rich signet ring adenocarcinomas, iii) signet ring adenocarcinoma – includes both mucin-rich and mucin-poor signet ring adenocarcinomas, iv) medullary carcinoma – tumors comprised of sheets of cells with vesicular nuclei, prominent nucleoli and abundant eosinophilic cytoplasm with prominent infiltrating lymphocytes.

The term **‘**undifferentiated carcinoma’ was not used in this study. It was difficult to subclassify medullary and undifferentiated carcinomas based on criteria outlined by the WHO. ^6^ 'Undifferentiated' is also a term used to describe the tumour Grade in those with less than 5% glandular differentiation. Therefore we refer to tumours with less than 5% glandular differentiation as 'Undifferentiated' by Grade and 'Medullary' by special type.

**Method Of Assessing Tumor Budding**

For the purposes of this study, all dMMR H&E stained post-surgical resection tumor histological sections were retrospectively reviewed for evidence of tumor budding using Nikon Eclipse 50i microscope. Rapid bud count method was used. ^7^ Tumor bud counts were generated in 5 regions at 200x magnification for each tumor slide. All tumor slides were examined. The scoring system was as follows: i) Score 0: indicates no evidence of budding, ii) Score 1 – refers to all cases with a budding score > 0 but less than one bud in at least 50% of fields examined, iii) Score 2 – cases with at least one bud in at least 50% of fields examined (also described as a median budding score of at least 1).

For example:

1. 5 tumour slides with scores of 0,0,0,0,0, each: tumour budding score 0
2. 6 tumour slides with scores of 0, 1, 0, 0, 1, 0: tumour budding score 1
3. 5 tumour slides with scores of 1, 0, 1, 1, 0: tumour budding score 2

In multivariable analyses cases with a median bud score of 1 (i.e. score 2) were classified as high budding, whereas those with a median bud score of <1 (i.e. score 0-1) were classified as low budding. ^7^

**Method Of Assessing PDC Grade**

PDC grade was assessed at the invasive front of the tumor under x200 magnification in a field containing maximum clusters. A PDC was defined as the presence of ≥5 cancer cells with no gland formation in a field containing maximum clusters. For this study we added a Grade 0 to that of the 3-tiered grading system of Ueno and colleagues. ^8^ The grading system was as follows: i) Grade 0 – the absence of PDCs, ii) Grade 1 - 1 to 4 PDCs, iii) Grade 2 – 5 to 9 PDCs, iv) Grade 3 – ≥ 10 PDCs. All tumor slides were assessed. The highest grade represented in at least two slides, was selected and the totals were added for a final PDC count.

For example:

1. 5 tumour slides with scores of 1, 2, 2, 3, 3 each: grade 3
2. 6 tumour slides with scores of 1, 1, 1, 2, 2, 3: grade 2
3. 5 tumour slides with scores of 0, 0, 1, 1, 3: grade 1

In multivariable analyses PDC Grade 0 – 1 were considered low PDC grade while PDC 2 – 3 were considered high PDC grade.

**Method Of Assessing Invasion Of Structures:**

The invasion of structures was assessed using the technique describe by Jaas. ^9, 10^ Extramural venous invasion (EMVI) refers to invasion of muscular vein by carcinoma. Lymphovascular invasion (LVI) refers to angiolymphatic invasion by carcinoma (i.e. tumour cells within endothelial-lined spaces). Perineural invasion (PNI) refers to the involvement of perineural spaces by carcinoma. All three features were described as either present or absent.

**Method Of Assessing The Tumour Border Configuration:**

The 2-tier Jass method of assessing the tumour border configuration was used. ^9, 10^ A percentage border configuration was estimated. An infiltrative border was characterised, on naked eye examination of a single microscopic slide of the tumor border, by an inability to define limits of invasive border of tumor and/or inability to resolve host tissue from malignant tissue. On microscopic examination an infiltitive border was identified by "streaming dissection" of muscularis propria (dissection of tumor through the full thickness of the muscularis propria without stromal response) and/or dissection of mesenteric adipose tissue by small glands, irregular clusters or cords of tumor cells and/or the presence of PNI. In contrast a pushing border was characterised by a smooth, expanding border. Tumours with > 50% pushing were described as having an expansile border while tumours with >50 infiltrative described as having an infiltrative border

**Method Of Assessing Clinical Outcomes**

Follow-up has been recorded through the prospectively maintained institutional colorectal cancer database and mortality status and cause of death was confirmed from data obtained from the National Cancer Registry, Cork, Ireland and the General Registrars Office, Dublin, Ireland. Primary care physicians were contacted as necessary to complete survival data if cause of death on the register was unclear.

**Statistical Analysis**

All results were analyzed using IBM SPSS Statistics for Mac OS, Version 21.0 (2012), IBM Corp, Armonk, New York, USA and GraphPad Prism for Mac OS, Version 7.0 (2016), GraphPad Software Inc, La Jolla California, USA.

All results were analyzed using IBM SPSS Statistics for Mac OS, Version 21.0 (2012), IBM Corp, New York, USA and GraphPad Prism for Mac OS, Version 7.0 (2016), GraphPad Software Inc, California, USA.

The Cohen’s kappa coefficient was used to test interobserver reliability. The statistical association between CDX2 status and the various histological parameters was investigated using Fisher’s Exact for categorical data as appropriate. The Independent Samples Student t test and Mann-Whitney U test were used for investigating the association between CDX2 status, and continuous variables. Univariable logistic regression analysis was used assess the association between each histological variable and the presence of LNM expressed in crude odds ratios (OR) with 95% confidence intervals (CIs). Variables with *P* < 0.200 in univariable analysis were included in the multivariable analysis. Multiple regression analysis using the forward conditional selection method was used to identify variables that contributed independently to the risk of LNM. Kaplan-Meier curves, the Log-rank (Mantel-Cox) test and Cox regression were used to associate survival with CDX2 status and the various molecular and pathological characteristics expressed as hazard ratios (HR) with 95% CIs. All tests of significance were two-tailed, with *P* < 0.050 indicating statistical significance.

**Ethical Approval**

The study protocol has been reviewed and approved by the Research and Ethics committee of St. Vincent’s University Hospital (SVUH); Elm Park, Dublin 4, Ireland (See attached documents). SVUH is one of the eight cancer centres in Ireland under the Health Service Executive National Cancer Control Program (NCCP) and the most recent report shows that approximately 400 patients were referred with CRC in 2014.^11^

**References**

1. RCSI. Clinical Guidelines Committee. Colorectal Cancer Management Clinical Guidelines. Dublin: Royal College of Surgeons in Ireland, 2002. . In; 2002.

2. Edge SB, Compton CC. The American Joint Committee on Cancer: the 7th edition of the AJCC cancer staging manual and the future of TNM. *Annals of surgical oncology* 2010;**17**(6): 1471-1474.

3. Mohan HM, Ryan E, Balasubramanian I, Kennelly R, Geraghty R, Sclafani F, Fennelly D, McDermott R, Ryan EJ, O'Donoghue D, Hyland JM, Martin ST, O'Connell PR, Gibbons D, Winter D, Sheahan K. Microsatellite instability is associated with reduced disease specific survival in stage III colon cancer. *Eur J Surg Oncol* 2016.

4. Dalerba P, Sahoo D, Paik S, Guo X, Yothers G, Song N, Wilcox-Fogel N, Forgo E, Rajendran PS, Miranda SP, Hisamori S, Hutchison J, Kalisky T, Qian D, Wolmark N, Fisher GA, van de Rijn M, Clarke MF. CDX2 as a Prognostic Biomarker in Stage II and Stage III Colon Cancer. *The New England journal of medicine* 2016;**374**(3): 211-222.

5. Hamilton SR VB, Kudo S, E. Riboli S, Nakamura P, Hainaut C.A, Rubio L.H, Sobin F. Fogt S.J, Winawer D.E, Goldgar J.R. Jass. Pathology and Genetics of Tumours of the Digestive System. In: IARC Press; 2010. WHO Classification of Tumours of the Digestive System. In. Lyon: IARC Press Lyon, 2000; 2000. p. 110-112.

6. Hamilton SR BF, Boffetta P. . *Carcinoma of the colon and rectum. In: IARC Press; 2010. WHO Classification of Tumours of the Digestive System. 4th ed.* IARC Press; 2010. p. 138-9.: Lyon, 2010.

7. Wang LM, Kevans D, Mulcahy H, O'Sullivan J, Fennelly D, Hyland J, O'Donoghue D, Sheahan K. Tumor budding is a strong and reproducible prognostic marker in T3N0 colorectal cancer. *The American journal of surgical pathology* 2009;**33**(1): 134-141.

8. Ueno H, Kajiwara Y, Shimazaki H, Shinto E, Hashiguchi Y, Nakanishi K, Maekawa K, Katsurada Y, Nakamura T, Mochizuki H, Yamamoto J, Hase K. New criteria for histologic grading of colorectal cancer. *The American journal of surgical pathology* 2012;**36**(2): 193-201.

9. Jass JR, Love SB, Northover JM. A new prognostic classification of rectal cancer. *Lancet (London, England)* 1987;**1**(8545): 1303-1306.

10. Jass JR. Classification of colorectal cancer based on correlation of clinical, morphological and molecular features. *Histopathology* 2007;**50**(1): 113-130.

11. St. Vincent’s Healthcare Group (SVHG) Cancer Report. <http://www.stvincents.ie/dynamic/File/SVHG-Annual-Cancer-Report-2015.pdf> [25/11/2016 2016].

**Fig. S1** Scoring system for CDX2 immunohistochemistry


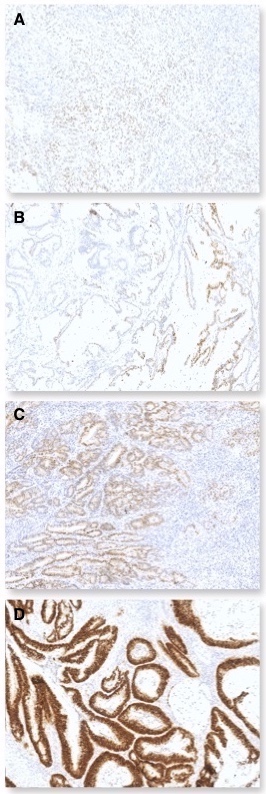

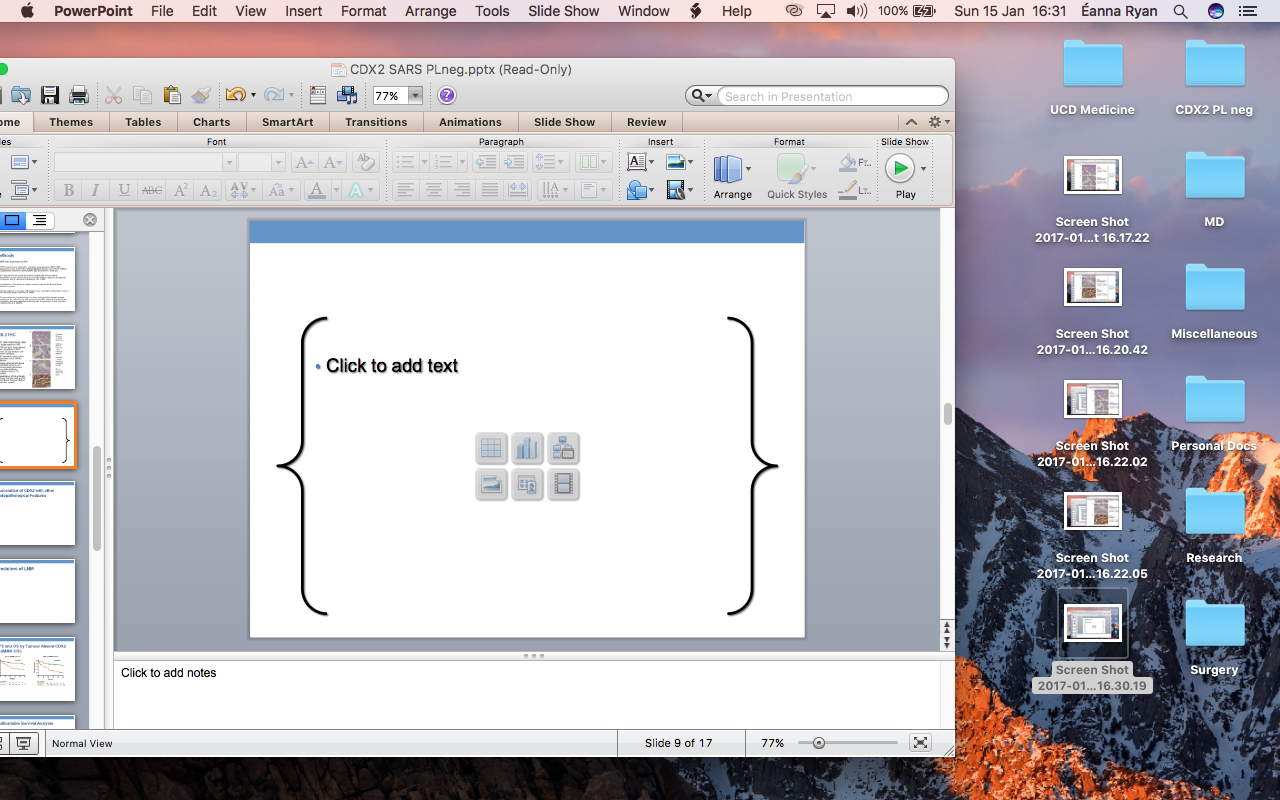


**Complete Absence of CDX2 expression (Score 0)**

Observed Frequency:

n = 14 (5.9%)


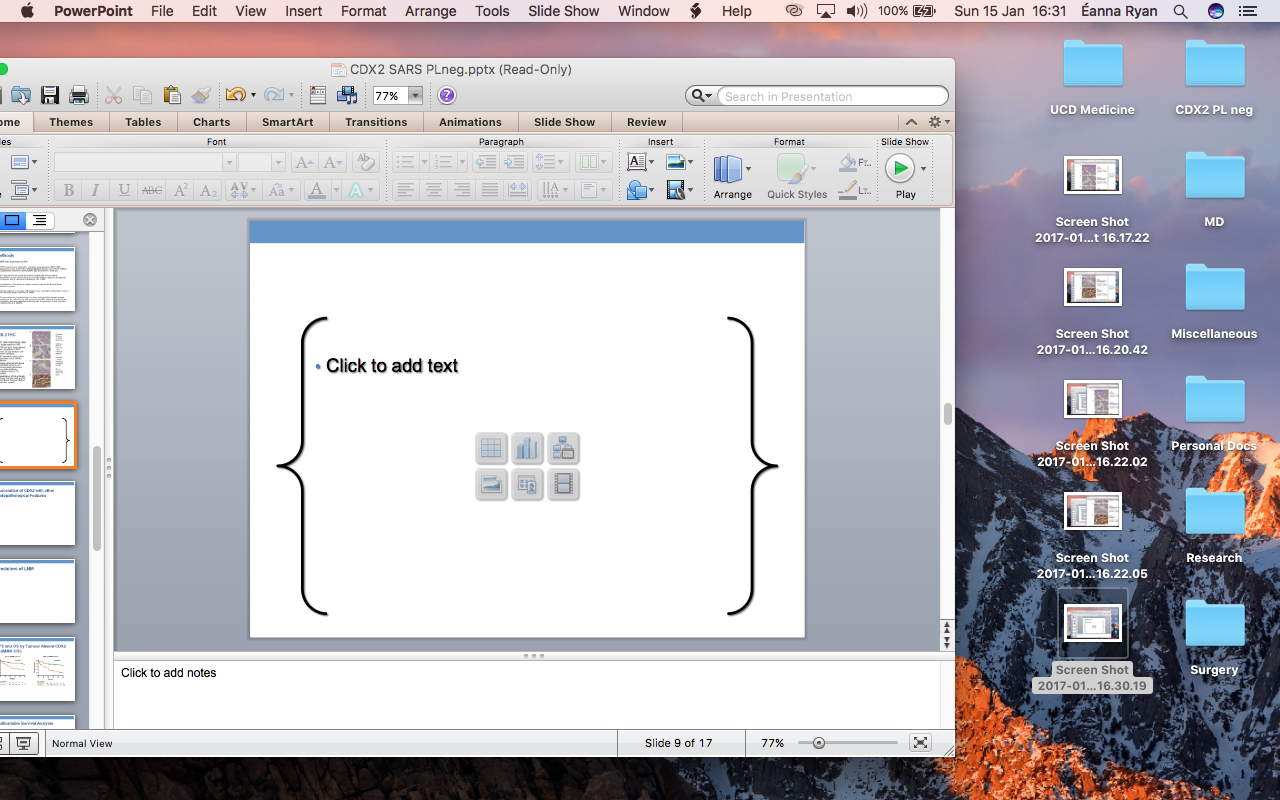


**CDX2 negative**

Observed Frequency:

n =37 (15.7%)

**CDX2 in a minority of cells (<20%) with weak/moderate staining (Score 0.5)**

Observed Frequency:

n = 23 (9.8%)

**κ = 0.782 (p<0.000)**

**Moderate/strong staining in a significant number of cells (Score 2)**

Observed Frequency:

n = 58 (24.7%)

**CDX2 positive**

Observed Frequency:

n = 198 (84.3%)

**Strong staining in all cells (Score 3)**

Observed Frequency:

n = 140 (59.6%)

**Fig. S2** Relationship between CDX2 protein expression, overall survival and nodal status in dMMR colorectal cancer


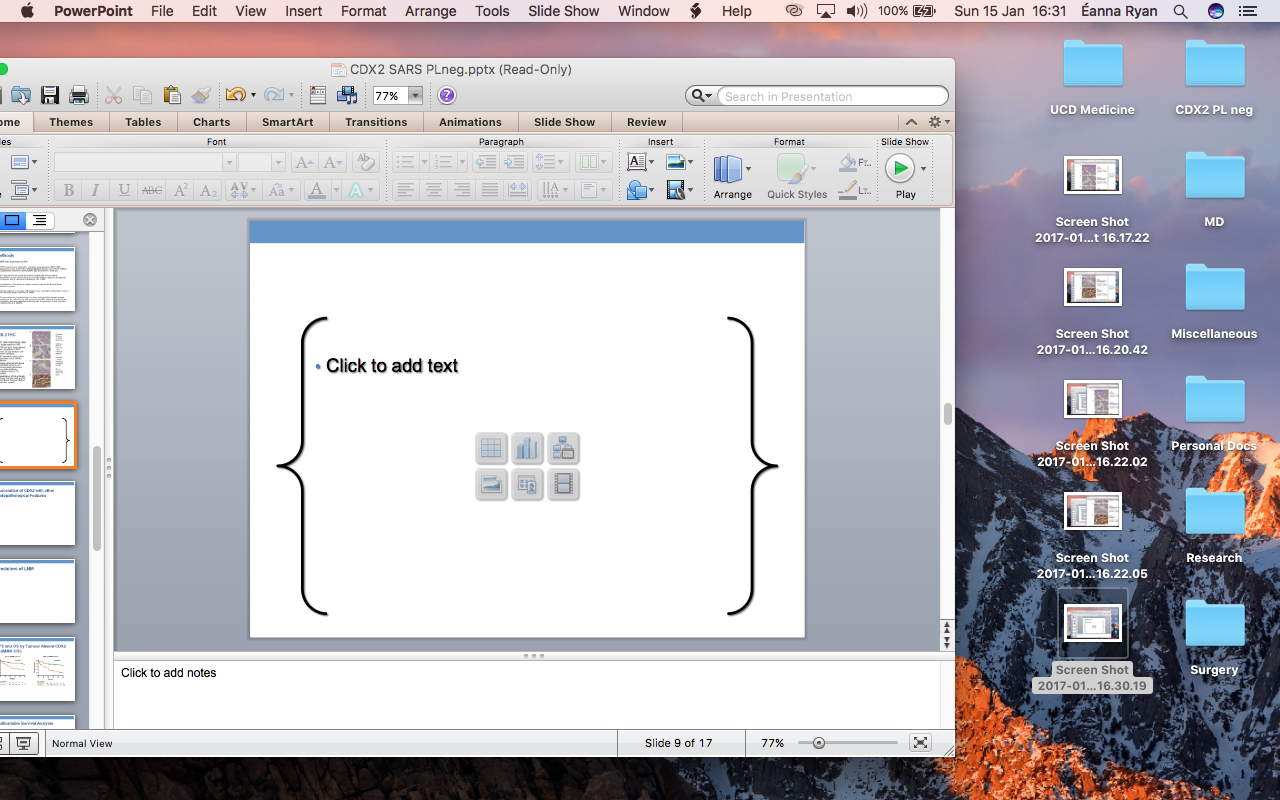
**A.** Stage-III tumors are enriched with specimens that lack CDX2 protein expression

**Pearson's Chi-squared Test**

**χ2 = 5.823**

***P* = 0.016***

| **Nodal status** | **CDX2 Status** | | **% CDX2neg** |
| --- | --- | --- | --- |
|  | **CDX2neg** | **CDX2pos** |  |
| **Node negative** | **20** | **146** | **12.0%** |
| **Node positive** | **17** | **52** | **24.6%** |

**B.** The prognostic effect of CDX2 expression for overall survival (OS) in dMMR CRC was not independent of stage

**Relationship between CDX2 expression, DFS and OS once adjusted for stage in dMMR CRC:** There was no significant difference in the 3-year and 5-year OS in the dMMR CRC cohort once adjusted for stage.

**Fig. S3** Relationship between CDX2 expression and benefit from adjuvant chemotherapy in stage II and stage III dMMR CRC


**Relationship between CDX2 expression and benefit from adjuvant chemotherapy in Stage-II and Stage-III patients:** To evaluate whether patients with CDX2neg tumors had benefited from adjuvant chemotherapy, we investigated the relationship between CDX2 status, 3-year and 5-year DFS and treatment with adjuvant chemotherapy in Stage-II/III patients (n=195) in dMMR CRC. We stratified patients according to CDX2 status (CDX2neg vs. CDX2pos) and compared the DFS of those treated with adjuvant chemotherapy with the DFS of those not treated with adjuvant chemotherapy (Chemo vs. No Chemo). Treatment with adjuvant chemotherapy was not associated with a statistically significant difference in 3-year survival in Stage-II/III dMMR CRC. However, treatment with chemotherapy was associated with a statistically significant reduction in 5-year DFS (DFS, Chemo vs. No chemo: 31.5% vs. 57.9%, *P* = 0.0264*). No benefit was observed in 3-year or 5-year DFS across the tested cohorts based on CDX2 expression.

**Table S1** Patient demographics and tumour histological features

| Parameter | Strata | n = (valid %) |
| --- | --- | --- |
| Mean Age in years +/- SD (Range) |  | 71.18 +/- 13.757 years (range 23 – 97) |
| Gender n= (%) | **Female** | 156 (65.5) |
|  | **Male** | 82 (34.5) |
| Median Size in mm (Range) |  | 50 mm (range 33 – 104) |
| Site n= (%) | **Right** | 200 (84) |
|  | **Left** | 38 (16) |
| AJCC 7^th^ TNM n= (%) | **1** | 37 (15.5) |
|  | **2** | 131 (55) |
|  | **3** | 64 (26.9) |
|  | **4** | 6 (2.5) |
| CDX2 Expression | **0** | 14 (5.9) |
|  | **0.5** | 23 (9.7) |
|  | **1** | 58 (24.4) |
|  | **2** | 140 (58.8) |
| BRAF | **Mutation +ve** | 144 (69.2) |
|  | **Wild Type** | 64 (30.8) |
| WHO grade n= (%) | **Low** | 149 (62.6) |
|  | **High** | 89 (37.4) |
| Budding | **Absent** | 130 (77.8) |
|  | **Present** | 37 (22.2) |
| PDC Grade | **Low** | 126 (75.4) |
|  | **High** | 41 (24.6) |
| Special Type | **Mucinous** | 38 (16.0) |
|  | **Signet Ring** | 19 (8.0) |
| LVI n= (%) | **Absent** | 107 (45.0) |
|  | **Present** | 131 (55.0) |
| PNI n= (%) | **Absent** | 204 (85.7) |
|  | **Present** | 34 (14.3) |
| EMVI n= (%) | **Absent** | 162 (68.1) |
|  | **Present** | 75 (31.5) |
| Margin | **Expansile** | 116 (48.9) |
|  | **Infiltrative** | 121 (51.1) |
| pT stage n= (%) | **pT1** | 8 (3.4) |
|  | **pT 2** | 32 (13.4) |
|  | **pT 3** | 93 (39.1) |
|  | **pT 4** | 105 (44.1) |
| pN stage n= (%) | **pN 0** | 168 (70.6) |
|  | **pN 1** | 45 (18.9) |
|  | **pN 2** | 25 (10.5) |
| pMstage n= (%) | **M0** | 232 (97.5) |
|  | **M1** | 6 (2.5) |
